# Multi-Sensor Data Fusion and CNN-LSTM Model for Human Activity Recognition System

**DOI:** 10.3390/s23104750

**Published:** 2023-05-14

**Authors:** Haiyang Zhou, Yixin Zhao, Yanzhong Liu, Sichao Lu, Xiang An, Qiang Liu

**Affiliations:** 1 Academy of Artificial Intelligence, Beijing Institute of Petrochemical Technology, Beijing 102617, China; 2 Beijing Academy of Safety Engineering and Technology, Beijing 102617, China

**Keywords:** human activity recognition, multi-sensor data fusion, fusion algorithm, CNN-LSTM

## Abstract

Human activity recognition (HAR) is becoming increasingly important, especially with the growing number of elderly people living at home. However, most sensors, such as cameras, do not perform well in low-light environments. To address this issue, we designed a HAR system that combines a camera and a millimeter wave radar, taking advantage of each sensor and a fusion algorithm to distinguish between confusing human activities and to improve accuracy in low-light settings. To extract the spatial and temporal features contained in the multisensor fusion data, we designed an improved CNN-LSTM model. In addition, three data fusion algorithms were studied and investigated. Compared to camera data in low-light environments, the fusion data significantly improved the HAR accuracy by at least 26.68%, 19.87%, and 21.92% under the data level fusion algorithm, feature level fusion algorithm, and decision level fusion algorithm, respectively. Moreover, the data level fusion algorithm also resulted in a reduction of the best misclassification rate to 2%~6%. These findings suggest that the proposed system has the potential to enhance the accuracy of HAR in low-light environments and to decrease human activity misclassification rates.

## 1. Introduction

In recent years, the progress in the areas of science and technology have resulted in improved living and medical conditions, leading to an increase in the average life expectancy of people. However, this increase has also made the problem of ageing more severe, particularly with regard to elderly home security. Due to the age-related decline in bodily function, the elderly are particularly susceptible to accidents in their daily lives, with falls being the leading cause of injury among this population. According to the World Health Organization [1], 42 percent of people over 70 are likely to fall at least once a year. The problem is exacerbated when elderly individuals live alone without supervision, as they may not receive timely treatment after a fall, which could potentially result in death. To address this issue, various sensors and technologies have been applied to monitor and recognize the activities of elderly individuals in their homes, with the aim of improving home safety through technical means [2]. Currently, camera-based methods are the mainstream approach for human activity recognition (HAR). This method has high accuracy and simple device deployment in normal light environments, and can effectively differentiate easily confused activities. However, in low-light environments, the recognition accuracy of cameras will significantly decrease. Furthermore, there is a higher likelihood of misclassification between different activities. The most critical issue with camera-based HAR methods is that they jeopardize the privacy of the monitored individuals in certain ways. Moreover, human activity recognition based on wearable sensors is another widely applied approach. While it can provide convenience for the monitored individual, its effectiveness is limited by the battery capacity of the devices and their ability to perform long-term uninterrupted monitoring. Furthermore, some wearable devices are too sensitive and may mistakenly identify certain daily activities as dangerous. Recently, there has been increasing interest in using millimeter-wave radar for HAR due to its ability to maintain performance even in low-light environments without being affected by light. However, the raw signal from millimeter-wave radar presents challenges in terms of processing, and millimeter-wave radar are generally more expensive than other types of sensors.

According to the characteristics of millimeter-wave radars and cameras, we have designed a multi-sensor HAR system by combining the stability and high accuracy of camera-based recognition in normal light with the ability of millimeter-wave radar to remain stable in low-light environments, regardless of light. The system can accurately recognize human activity in both normal light and low-light environments, while also reducing the misclassification rate of certain confusing activities. Our method also focused on the fusion of camera and millimeter wave radar data to address the limitations inherent in single-sensor systems, enabling the HAR system to achieve the efficient and accurate recognition of human activity across a range of light environments. Our work involved theoretical analysis, model building, algorithm development, data collection, and experimental verification to accomplish this goal.

## 2. Related Work

Numerous researchers have extensively studied HAR from various angles, including the use of sensors and algorithms. Currently, the focus of HAR research has shifted from direct recognition of physical information captured by sensors to artificial intelligence-based recognition through machine or deep learning after extracting relevant human motion features from collected data. Wearable devices, cameras, and millimeter-wave radar are three commonly used devices in HAR. Furthermore, with advancements in sensor and algorithm technologies, researchers have explored various fusion systems for HAR.

### 2.1. HAR Based on Single Sensor Data

HAR based on camera and computer vision technology is a widely used method. Some researchers [3,4] have utilized background subtraction to extract features with deep learning methods after separating the image background from the human. Compared with manual feature extraction methods, deep learning methods can express features more abstractly and possess better generalization and robustness. Nunez-Marcos et al. [5] employed optical flow image sequences as inputs to a convolutional network to ensure that the features were independent of the background and without interference. Feichtenhofer et al. [6] proposed spatial fusion and temporal fusion methods to improve HAR accuracy, achieving 93.5% and 69.2% in UCF101 and HMDB51, respectively. However, since video sequences encompass both spatial and temporal features, while a single CNN model may lose temporal features, some researchers [7,8,9] have proposed model combination methods to enhance the accuracy of HAR. For example, Zhang et al. [10] combined two CNNs to form a 2D-CNN network to obtain the spatial and temporal features of video sequences, achieving an accuracy of 90.9% on the NTU-RGB+D dataset. Chadia Khraief et al. [11] constructed a model with four independent CNNs corresponding to video data and utilized data combined with a 4D-CNN network to verify its effect on several datasets. Nandagopal et al. [12] designed a novel key point extraction with a deep convolutional neural network-based pose estimation (KPE-DCNN) model to extract the key points of the human body in the image sequences converted from video data for HAR. The KPE-DCNN model outperforms other networks, such as CNN, DBN, and T-CNN, achieving an accuracy of 85.44% on the UCF dataset. Generally, HAR based on video or image sequences has relatively high recognition accuracy and robustness. However, the performance of this method will decline rapidly in low-light environments. The utilization of camera-based monitoring systems for HAR continues to pose a challenge, especially in instances where activity identification is hindered by the absence of individuals in images captured within low-light settings. To address this issue, researchers have generally focused on two areas: algorithms and sensors. Current research has utilized both near-infrared (NIR) and long-wavelength infrared (LWIR) cameras to tackle this issue. Compared to NIR, LWIR cameras can detect objects at both long and short distances without requiring additional light, whereas NIR cameras require extra light to display only short-distance objects in low-light environments [13]. However, there are two main drawbacks of thermal imaging cameras: the halo effect and temperature similarity. The halo effect occurs around high-temperature objects. In human bodies, this type of halo effect is similar to shadows under body areas. This means that it is more difficult to segment the human area from images with halo effects. To address this issue, Batchuluun et al. [14] considered a method for creating skeletal images from thermal images to analyze body movements. In order to extract more spatial and temporal features from the resulting sequence of skeletal images, they proposed a HAR method that combines CNN and LSTM to improve recognition accuracy. Some researchers solved the problem by using HAR algorithms. In this paper [15], a novel procedure is produced to model, analyze, and recognize human motion (jogging and walking in a dark environment) in video streams. They use an image processing technique using edge detection and vector angle calculation to improve accuracy. In many cases, the use of multi-sensor video streams to improve accuracy is encouraged. However, recognizing activities from multiple video streams simultaneously is a challenge due to their complementarity and noise. Ulhaq [16] proposed the use of deep multi-view representation learning for simultaneous HAR from multiple video streams. Additionally, they also introduce a spatio-temporal feature-based correlation filter for detecting and recognizing multiple human activities under low-light environments. Although these improved algorithms and technologies applied to sensors can reduce noise in image or video stream data in low-light environments, thus improving the accuracy of HAR, the accuracy of a camera in low-light environments is limited by its own characteristics. Moreover, camera-based data collection is unsuitable for specific environments such as bathrooms, bedrooms, and other private areas, violating personal privacy and leading to moral and legal issues [17].

With the growing privacy concerns around cameras, HAR based on millimeter-wave radar data has emerged as a promising solution. The key challenge in using millimeter-wave radar data for HAR is to extract and identify the micro-doppler signal features generated by human motion. In recent years, several researchers have proposed various approaches to address this challenge. For instance, Liu et al. [18] utilized MFCC (Mel-frequency cepstrum coefficients) to extract the time-frequency features of walking, bending, falling, and other activities. They further employed the SVM (Support Vector Machine) and KNN (K-Nearest Neighbor) algorithms to classify and recognize these activities. Jokanovic et al. [19] converted the spectrogram of walking, falling, sitting, and bending into grayscale images and used DNNs (Deep Neural Networks) and SVM to recognize them, reaching an accuracy of 87% and 78%, respectively. In another study, Erol et al. [20] proposed a sequence forward selection method combined with SVM reclassification according to the different number of features used. The approach achieved an accuracy of between 92% and 95%. Sadreaza et al. [21] and Tsuchiyama et al. [22] combined the time-distance spectrogram and time series of radar data with CNN (Convolutional Neural Network) for HAR. Bhattacharya et al. [23] used a low-cost, ceiling-mounted radar system at low microwave frequencies (sub-6GHz), which was combined with a small neural network model to detect breath and distinguish falls from non-falls with an accuracy of 95%. Furthermore, Wang et al. [24] proposed an improved LSTM (long short-term memory) model based on an RNN (Recurrent Neural Network). They combined this model with deep CNN to recognize Doppler radar images of six types of human activities with an accuracy of 82.33%. Despite its effectiveness, the accuracy of a single millimeter-wave radar system is still challenging in terms of achieving practical applications. The performance of millimeter-wave radars is not affected by light, but it degrades significantly in noisy environments, and the accuracy is lower than that of cameras in normal light.

### 2.2. HAR Based on Multi-Sensor Data Fusion

With advances in technology, HAR based on multi-sensor data fusion has gained attention from researchers. Cornacchia et al. [25] utilized accelerometer, pressure sensor, and gyroscope sensors combined with a camera to recognize human activities. The wearable sensors were used to extract global activity features, while the camera was used to extract local activity features. Shoaib et al. [26] designed a wrist-worn multi-sensor motion recognition system to distinguish repetitive activities such as walking and jogging from less-repetitive activities, such as smoking and giving a talk. Brezmes et al. [27] used a smartphone combined with infrared sensors in the room to identify human poses and motion states. Most methods for combining multiple sensor data to recognize human activity are based on three fusion algorithms: data level fusion, feature level fusion, and decision level fusion. Capela et al. [28] used the data level fusion algorithm to combine different sensors. However, this method requires a large bandwidth and significant power consumption due to the need to transmit a large amount of raw data from wearable devices. LI Kuncheva [29], Min and Cho [30] used the feature level fusion algorithm to cascade the features of multiple different sensor data after feature extraction, and then utilized machine learning algorithms for HAR. However, this method may not be convenient to cascade the data generated by different sensors with different sampling frequencies, and it may also ignore the contribution of some sensors to the results of recognition. LeCun et al. [31] utilized the decision level fusion algorithm to fuse the data from the accelerometer and the heart rate sensor for HAR. However, traditional machine learning algorithms only fuse the classification results and ignore the correlation between the data features of different sensors, which may not improve the results. Although these fusion algorithms combined with different sensors can recognize some simple human activities, challenges still exist in effectively distinguishing human activities in low light environments. Therefore, in this paper, we aim to improve the accuracy of HAR in low light environments by combining the advantages of camera and millimeter-wave radar through multi-sensor data fusion and an improved CNN-LSTM model. Our method does not require too much additional data processing to extract human activity features. In addition, the system can only set up millimeter-wave radar in private spaces.

## 3. System Design

**Figure 1:**
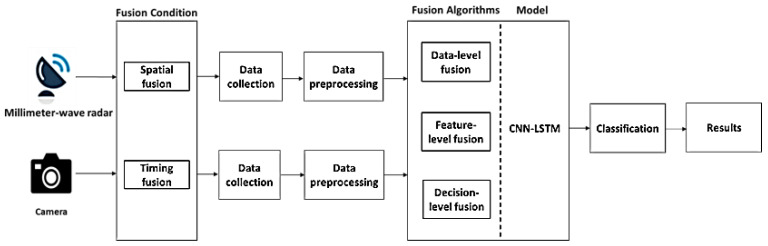
The block diagram of the multi-sensor system.

In this paper, we present a multi-sensor system for HAR that addresses the limitations of camera-based systems in low-light environments. Our system leverages millimeter wave radar and camera data, which are calibrated through spatiotemporal fusion techniques to improve accuracy. To achieve accurate HAR, our system implements sensor calibration through spatiotemporal fusion. We then collect different types of human activity data through sensors and preprocess them to make them suitable for input to our model. Finally, we use a proposed CNN-LSTM model, combined with various fusion algorithms, to classify the data and output the final results. Figure 1 illustrates the block diagram of our multi-sensor system.

### 3.1. System Construction

In our multi-sensor system, the camera continuously captures images of human activities, which serve as inputs to the model. The resolution of the camera significantly impacts image quality, and low-resolution images can result in unsatisfactory results. Conversely, high-resolution cameras can lead to better model training results but require longer data processing times. To strike a balance between image quality and processing time, we chose the Logi C270 USB camera with a resolution of 1280 × 720 and a maximum frame speed of 30 frames/s. This camera meets our requirements for HAR recognition and offers strong versatility. Additionally, it allows for direct data transmission to the computer via a USB interface, enabling real-time data processing. Table 1 presents the parameters of the Logi C270 USB camera.

The millimeter wave radar is capable of recognizing human activities by extracting and identifying micro-Doppler signal features generated during human motion. As the emitted electromagnetic wave and detected target have relative movement, the frequency of the echo differs from that of the emitted wave. This frequency difference can be detected to measure the moving speed of the target relative to the radar, while the distance of the target can be determined by measuring the time difference between pulse transmission and reception. Currently, many countries and regions utilize 60GHz millimeter wave radar for indoor personnel detection and SLAM routing. In this paper, we employed the Texas Instruments (TI) 60GHZ IWR6843ISK millimeter wave radar in combination with DCA1000EVM and MMWAVEICBOOST to collect human activity data. The DCA1000EVM provides access to raw radar data via the LVDS interface, while the MMWAVEICBOOST supports software development and tracking capabilities.

(1) Information about the IWR6843ISK

The IWR6843ISK is a millimeter wave sensor evaluation board produced by TI. The board uses TI’s IWR6843AW chip and integrates components such as antennas, RF front-end, and processors for measuring and detecting distance, velocity, and direction information. Components and parameters of the IWR6843ISK are shown in Table 2 and Table 3.

(2) Information about DCA1000EVM

DCA1000EVM is a digital signal processor used in RF receivers for radar applications. TI provides GUI software for configuring and controlling DCA1000EVM, as well as MATLAB scripts for data processing and visualization.

(3) Information about MMWAVEICBOOST

TI’s MMWAVEICBOOST is a millimeter-wave radar sensor module that is used for high-precision environmental sensing and ranging functions. The specifications and performance parameters of MMWAVEICBOOST are shown in Table 4.

**Figure 2:**
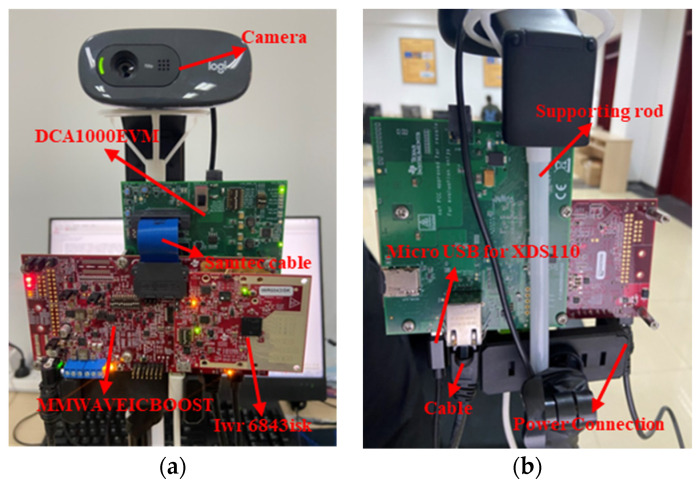
The multi-sensor system. The appearance of the system’s front and back. (**a**) The appearance of the front of the system. (**b**) The appearance of the back of the system.

In order to establish a multi-sensor system and minimize data discrepancies resulting from sensor location differences, we positioned the camera and millimeter radar on the same vertical line. Specifically, the millimeter-wave radar was fixed 150 cm above the ground, while the camera was placed 10 cm above it. Both sensors were connected to a computer via USB, and data collection and processing were carried out using Python code running on the computer. Figure 2 depicts the setup of the multi-sensor system.

### 3.2. System Calibration

System calibration is an essential step in fusing different sensor data. Due to the varying locations of sensors, the data coordinates obtained by different sensors are different. Hence, it is critical to remap the spatial relationship between different sensors into the world coordinate system using spatial calibration. Furthermore, when different sensors collect data at differing frequencies, it is also necessary to match and calibrate the time of each sensor to enable the time fusion of multiple sensors.

#### 3.2.1. Spatial Calibration

**Figure 3:**
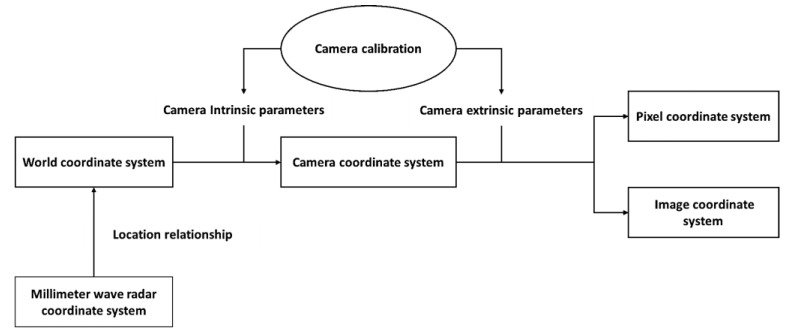
The spatial relationship between the coordinate systems.

To achieve spatial calibration, the coordinate systems of different sensors need to be transformed, since their positions cannot completely coincide. The resulting spatial coordinate information is then mapped onto the world coordinate system. Figure 3 illustrates the spatial relationship between the coordinate systems of the millimeter wave radar and camera.

(1) Transformation of the camera coordinate system

**Figure 4:**
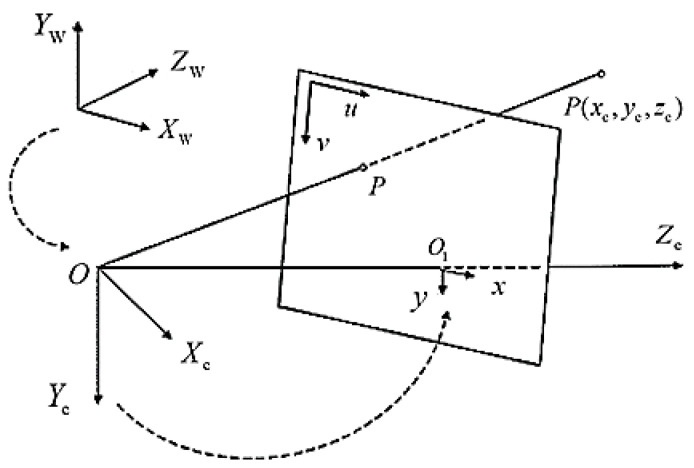
The relationship between the four coordinate systems.

Assuming that the world coordinate system is represented as $\left(X_{W},Y_{W},Z_{W}\right)$ and the camera coordinate system as $\left(X_{C},Y_{C},Z_{C}\right)$, where $Z_{C}$ denotes the axis of the light emanating from the camera. In addition, the image coordinate system is (x, y), and the pixel coordinate system is (u,v). Figure 4 illustrates the relationship among these four coordinate systems.

Assuming that the human object is represented by a point, P, the method of calculating its transformation from world coordinates to camera coordinates is as follows.

(1) $$\left[\begin{array}{c} X_{C}\\ Y_{C}\\ Z_{C}\\ 1 \end{array}\right]=\left[\begin{array}{cc} R & T\\ 0 & 1 \end{array}\right]\left[\begin{array}{c} X_{W}\\ Y_{W}\\ Z_{W}\\ 1 \end{array}\right]$$

*R* represents a 3 × 3 rotation matrix, while T is a three-dimensional translation vector.

Based on the principle of pinhole imaging, the point P $\left(x_{c},y_{c},z_{c}\right)$ in camera coordinates can be transformed into image coordinates using Equation (2):

(2) $$x=\frac{fX_{C}}{Z_{C}},y=\frac{fY_{C}}{Z_{C}}$$

in Equation (2), f represents the focal length.

By transforming Equation (2) into homogeneous coordinates, we obtain the following equation:

(3) $$Z_{C}\left[\begin{array}{c} x\\ y\\ 1 \end{array}\right]=\left[\begin{array}{cccc} f & 0 & 0 & 0\\ 0 & f & 0 & 0\\ 0 & 0 & 1 & 0 \end{array}\right]\left[\begin{array}{c} X_{C}\\ Y_{C}\\ Z_{C}\\ 1 \end{array}\right]$$

**Figure 5:**
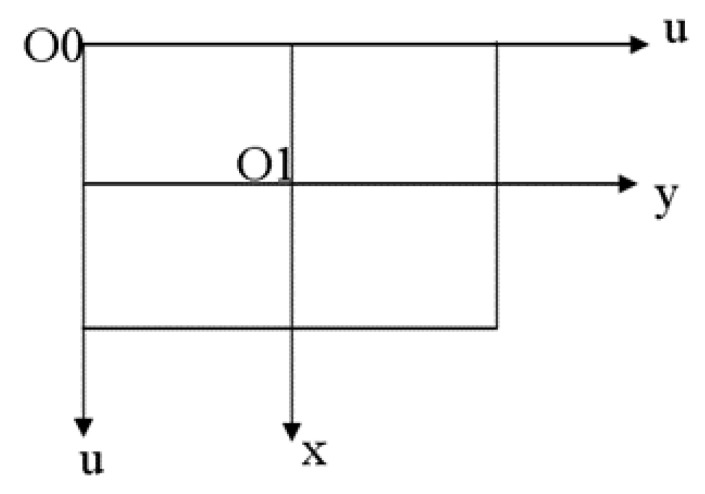
The relationship between the image and pixel coordinate.

If the point of ($u_{o},v_{o}$) represents the pixel coordinate, the relationship between the image and pixel coordinate is shown in Figure 5.

The relationship between the two coordinate systems can be expressed as a matrix:

(4) $$\left[\begin{array}{c} u\\ v\\ 1 \end{array}\right]=\left[\begin{array}{ccc} \frac{1}{dx} & 0 & -u_{0}\\ 0 & \frac{1}{dy} & v_{0}\\ 0 & 0 & 1 \end{array}\right]\left[\begin{array}{c} y \end{array}\right]$$

Equation (5) can be obtained by inverse transformation:

(5) $$\left[\begin{array}{c} x\\ y\\ 1 \end{array}\right]=\left[\begin{array}{ccc} dx & 0 & -u_{0}dx\\ 0 & dx & v_{0}dy\\ 0 & 0 & 1 \end{array}\right]\left[\begin{array}{c} u\\ v\\ 1 \end{array}\right]$$

The mapping of the world coordinate to the pixel coordinate is as follows:

(6) $$Z_{C}\left[\begin{array}{c} x\\ y\\ 1 \end{array}\right]=\left[\begin{array}{ccc} \frac{1}{dx} & 0 & -u_{0}\\ 0 & \frac{1}{dy} & v_{0}\\ 0 & 0 & 1 \end{array}\right]\left[\begin{array}{cccc} f & 0 & 0 & 0\\ 0 & f & 0 & 0\\ 0 & 0 & 1 & 0 \end{array}\right]\left[\begin{array}{cc} R & T\\ 0 & 1 \end{array}\right]\left[\begin{array}{c} X_{W}\\ Y_{W}\\ Z_{W}\\ 1 \end{array}\right]$$

Finally, we derive Equation (6) to get Equation (7).

(7) $$Z_{C}\left[\begin{array}{c} x\\ y\\ 1 \end{array}\right]=\left[\begin{array}{cccc} a_{x} & 0 & u_{0} & 0\\ 0 & a_{y} & v_{0} & 0\\ 0 & 0 & 1 & 0 \end{array}\right]\left[\begin{array}{cc} R & T\\ 0 & 1 \end{array}\right]\left[\begin{array}{c} X_{W}\\ Y_{W}\\ Z_{W}\\ 1 \end{array}\right]$$

$\left[\begin{array}{cccc} a_{x} & 0 & u_{0} & 0\\ 0 & a_{y} & v_{0} & 0\\ 0 & 0 & 1 & 0 \end{array}\right]$ is the intrinsic matrix of the camera and $\left[\begin{array}{cc} R & T\\ 0 & 1 \end{array}\right]$ represents the extrinsic matrix of the camera.

**Figure 6:**
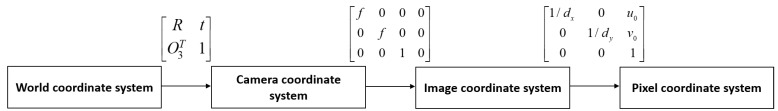
Mapping process of camera coordinate system.

The process of mapping spatial information from the camera to the world coordinate system is illustrated in Figure 6.

**Figure 7:**
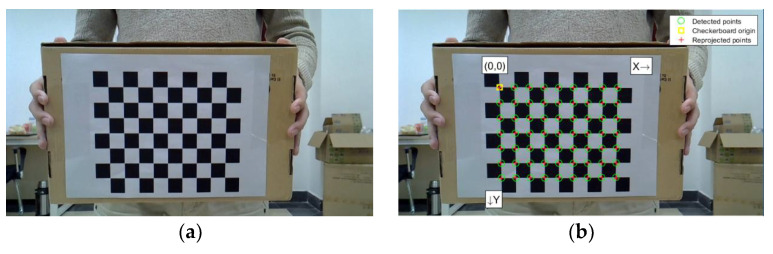

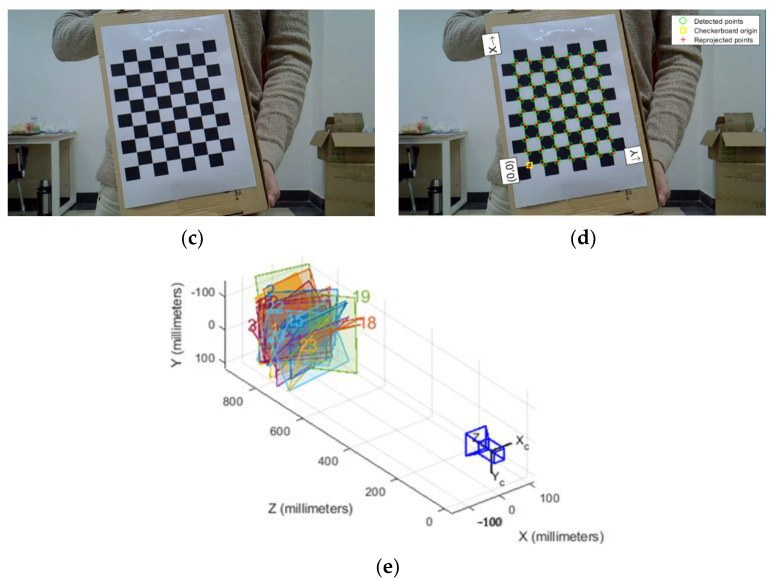
Calibration map and 3D calibration space centered on the camera. (**a**) Map 1 before calibration. (**b**) Map 1 after calibration. (**c**) Map 2 before calibration. (**d**) Map 2 after calibration. (**e**) 3D calibration space.

For our work, we captured 24 calibration maps at different angles to calibrate the camera coordinate system [32]. These calibration maps were calibrated using MATLAB functions, with each cell in the map measuring 25 mm×25 mm. Figure 7a through Figure 7d display the calibration maps, whereas Figure 7e shows the 3D calibration space centered around the camera.

After the analysis, we can directly obtain the parameters of the camera. The parameters are given in the following Equations (8)–(10):

Intrinsic matrix:

(8) $$K=\left[\begin{array}{ccc} 802.151 & 0 & 307.383\\ 0 & 802.318 & 205.909\\ 0 & 0 & 1 \end{array}\right]$$

Rotation matrix:

(9) $$R=\left[\begin{array}{ccc} 0.2216 & -0.05552 & 0.0252\\ 0.9931 & 0.00089 & -0.7672\\ 0.1146 & 0.21703 & -0.7721 \end{array}\right]$$

Translation vector:

(10) $$T=\left[\begin{array}{ccc} -0.3827 & 0.2561 & 13.5561 \end{array}\right]$$

(2) Transformation of the Millimeter Wave radar coordinate system

**Figure 8:**
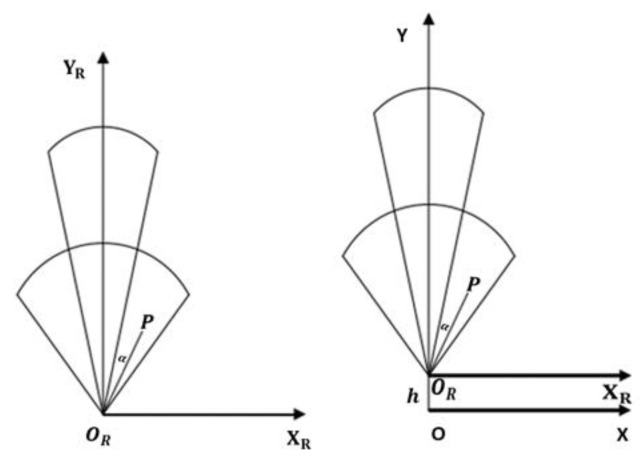
The transformation of the Millimeter Wave radar coordinate system.

Millimeter-wave radar data and camera data are collected in different coordinate systems, so we must transform the millimeter-wave radar coordinates into world coordinates. This is assuming that the millimeter-wave radar has a coordinate system of $\left(X_{R},O_{R},Y_{R}\right)$, while the world coordinate system is (X,O,Y) and the camera is located h units above the radar. The distance between the target P and the system is R, with an angle α between them. We positioned the millimeter-wave radar and the camera along the same straight line, such that the system is perpendicular to the plane where the target is located, resulting in $Y_{R}=Y$. Figure 8 illustrates the transformation of millimeter wave radar coordinate system.

The formula for mapping the target P from the millimeter-wave radar coordinates to the world coordinates is given in Equation (11).

(11) $$\{\begin{array}{c} X=R\times \sin\alpha \\ Y=h+R\times \cos\alpha \end{array}$$

#### 3.2.2. Time Calibration

**Figure 9:**
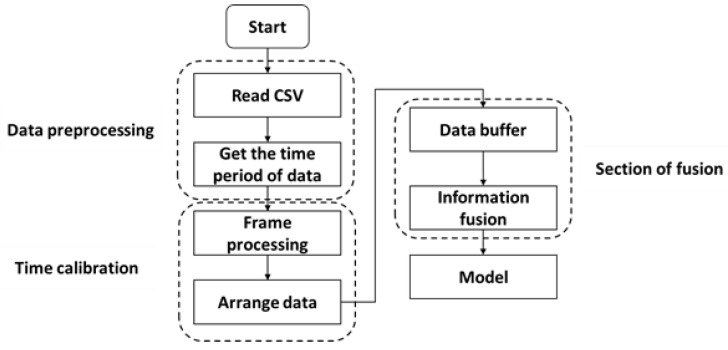
The process of time calibration.

Time calibration is an essential step for multi-sensor data fusion due to the inconsistent data collection frequency among different sensors. Specifically, the millimeter-wave radar collects data at a rate of 50 ms/frame, while the camera has a collection speed of 30 frames/s. As the collection rate of the camera is much higher than that of the millimeter-wave radar, it becomes necessary to downsample the camera data to match the time series of the millimeter-wave radar data collection. The process of time calibration is shown in Figure 9.

The following are the steps involved in time calibration:

1. Reading the CSV data.
2. Obtaining the timestamp of each sensor.
3. Reducing the frame rate of the camera from 30 frames/s to 20 frames/s.
4. Aligning the timestamp of the camera data with that of the radar data.
5. Data fusion.

### 3.3. Data Preprocessing

#### 3.3.1. Millimeter-Wave Radar Data

**Figure 10:**
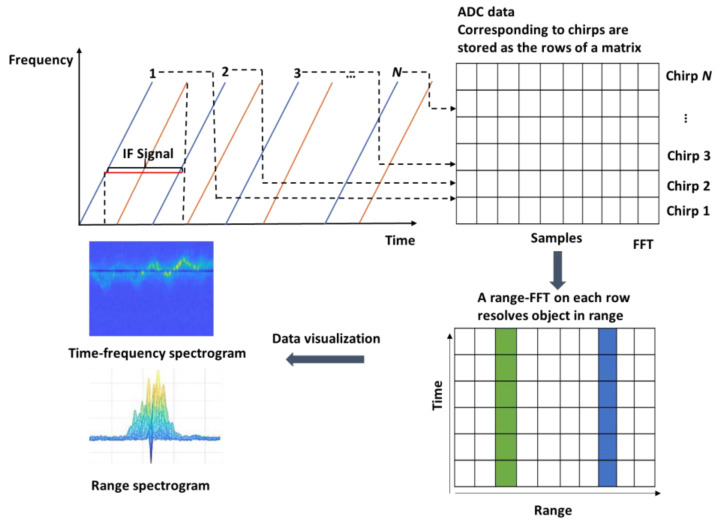
Spectrogram visualisation.

We collect radar data using the IWR6843, which has a bandwidth of 4 GHz, a chirp duration of 100 μs, and an output power of 12 dBm. The radar can record the micro-Doppler signals of moving people in the region of interest, and the format of each collected original radar data is a long 1D complex array. However, the 1D array signal is not suitable to be directly input into the model for training, so it needs to be preprocessed. As shown in Figure 10, the radar system transmits a chirp signal and receives a reflected chirp signal to produce an intermediate frequency (IF) signal. ADC sampling is carried out on the IF signal, and then Fast Fourier transform (FFT) is used to extract the frequency information of the signal. Fourier transform processing results in a frequency spectrogram that has separate peaks denoting the presence of an object at a specific distance. After FFT, we get the range, so this process is called Range-FFT. In order to better reflect the features of human activities through Range-FFT results, we visualize the Range-FFT results as time-frequency spectrograms and Range spectrograms.

(1) Time-frequency spectrogram

In this work, we convert the 1D millimeter wave radar signal into a two-dimensional (2D) time-frequency spectrogram with STFT (Short-time Fourier Transform) and provide it in Equation (12).

(12) $$STFT_{f}\left(t,f\right)=\int_{-\infty }^{\infty }f\left(t\right)\eta^{*}\left(t^{\prime }-t\right)e^{-2\pi ft}dt$$

f(t) is the target signal which we want to transform, and η(t) is the window function applied to the target signal.

**Figure 11:**
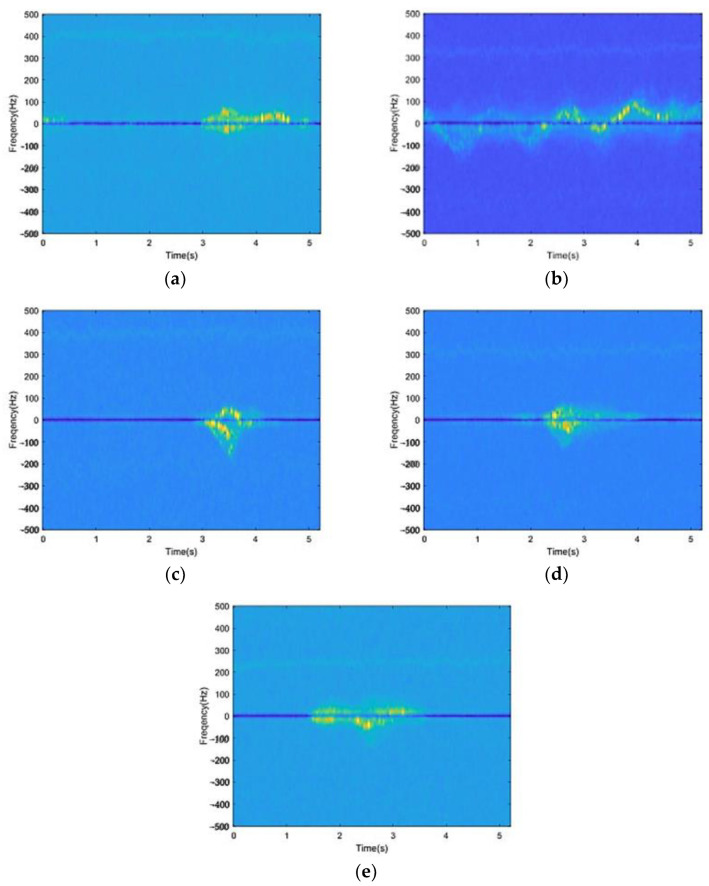
Time-frequency spectrogram. Frequency spectrograms of five activities after STFT transformation. (**a**) Sitting. (**b**) Walking. (**c**) Bending. (**d**) Squatting. (**e**) Falling.

The typical radar time-frequency spectrogram of five activities is shown in Figure 11.

(2) Range spectrogram

**Figure 12:**
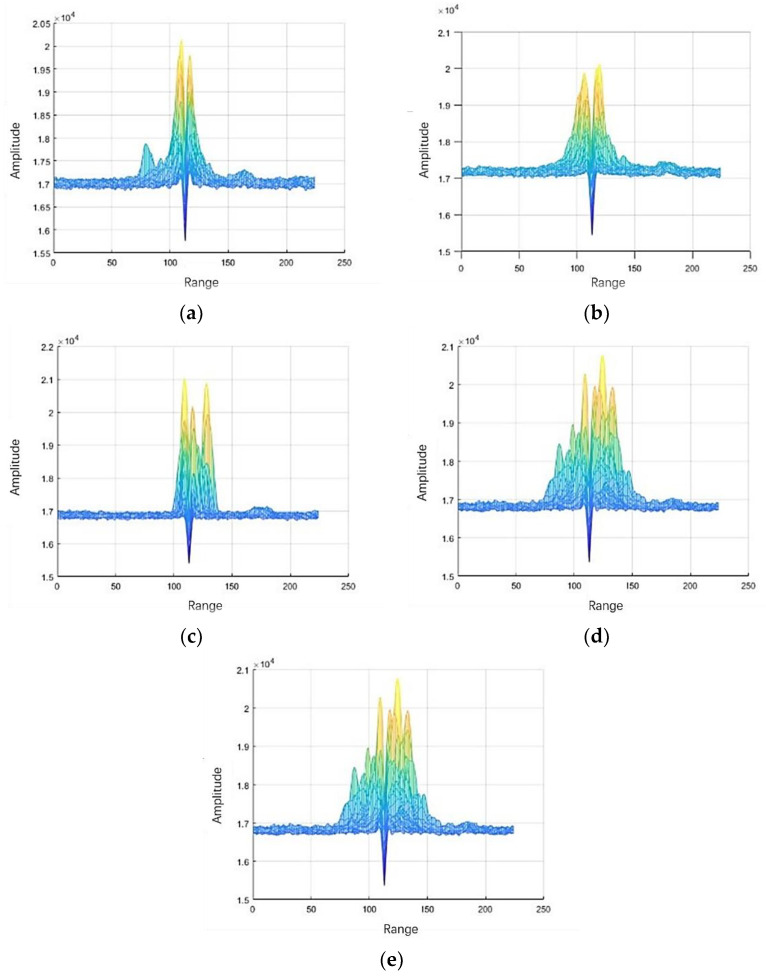
Range spectrogram. (**a**) Sitting. (**b**) Walking. (**c**) Bending. (**d**) Squatting. (**e**) Falling.

Since human activities will also cause changes in distance, we can extract the motion features of different human activities through the range spectrogram, which reflects the changes in distance, as shown in Figure 12.

(3) Noise reduction

Due to interference in the collection environment, the spectrogram of radar data often contains noise. The commonly used image denoising methods are spatial filtering, temporal accumulation, and machine learning and deep learning reconstruction. In this paper, we use 2D median filtering to reduce noise in the spectrogram. The principle of median filtering is to replace the value of a point in a digital image or digital sequence with the median value of each point in the neighborhood of that point. The equation for the median filter is shown in (13).

(13) $$y_{i}=Med\{f_{i-v},\ldots \ldots ,f_{i},\ldots \ldots ,f_{i+v}\},\;i\in N,v=\frac{m-1}{2}\;$$

**Figure 13:**
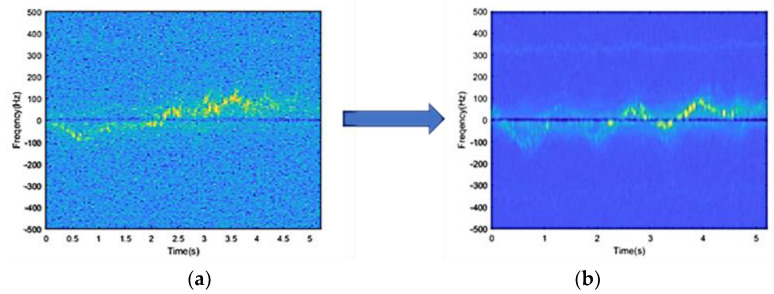
Noise reduction of a walking spectrogram. (**a**) Spectrogram before noise reduction. (**b**) Spectrogram after noise reduction.

This method can change the pixel with large differences in the surrounding gray value to a value close to the surrounding pixel value, thus reducing the noise points. The spectrogram before and after noise reduction is shown in Figure 13a,b.

#### 3.3.2. Video Data

**Figure 14:**
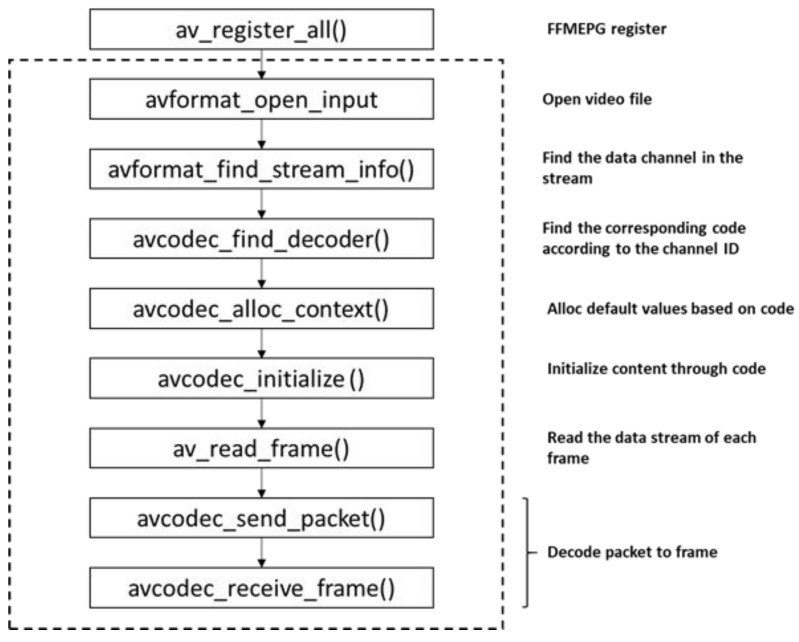
The process of video data conversion.

In this work, we utilized OpenCV for video recording. The collection frame rate was 30 frames/s, and the video collection window size was 640 pixels. The duration of each video recording was 3 s. We recorded five types of human activities: sitting, walking, bending, squatting, and falling under two lighting environments: normal light and low light. Since a video is essentially a sequence of images (referred to as frames) captured and eventually displayed at a given frequency, we employed FFMPEG software to convert the video data into individual image sequences. The process of video data conversion is visualized in Figure 14.

**Figure 15:**
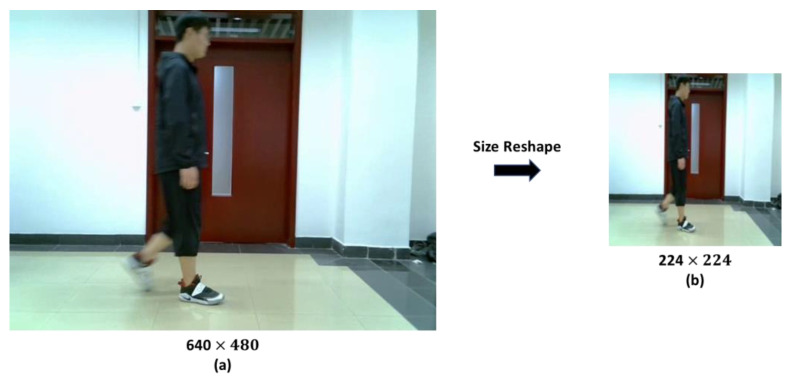
The result of the picture size reshape. The diagram is divided into two parts, which show the process of cropping the original size image to the size required by the model input. (**a**) The original size image. (**b**) The reshaped size picture.

Once the video data was converted to image sequences, we resized the image sequences and normalized it to match the requirements of the deep learning model input. The original size of each image was 640 × 480 pixels. After scaling the image size, it was changed to 224 × 224 pixels. The result of the picture size reshaping is shown in Figure 15.

**Figure 16:**
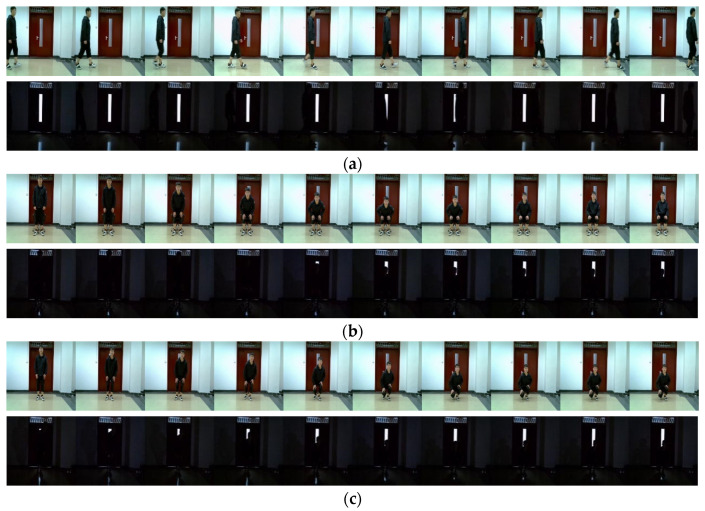

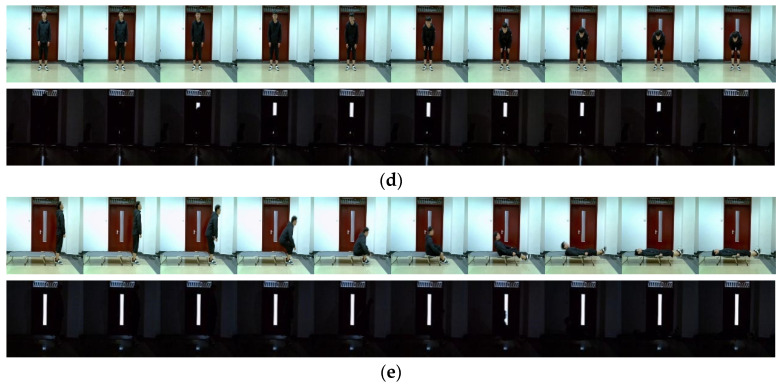
Image sequence in normal and low-light environments. The combination of the image sequence of five activities under normal and low light environments, respectively. (**a**) Sitting. (**b**) Walking. (**c**) Bending. (**d**) Squatting. (**e**) Falling.

In order to ensure that the frame number of the two types of data matched during model training, we reduced the frame number of the equispaced image sequence, while keeping the timing features of the image sequences as much as possible. Ultimately, we chose 20 frames for both the millimeter-wave radar data and the image sequences, which were used as inputs for the model. Figure 16a–e show a part of the image sequences captured under normal and low-light environments.

### 3.4. Model Design

We designed a combined CNN-LSTM network based on a convolutional neural network (CNN) and long short-term memory (LSTM). The CNN was used to extract the spatial features of the data, while the LSTM was used to model the temporal feature vector and extract the temporal features.

#### 3.4.1. Combined CNN-LSTM Network

**Figure 17:**
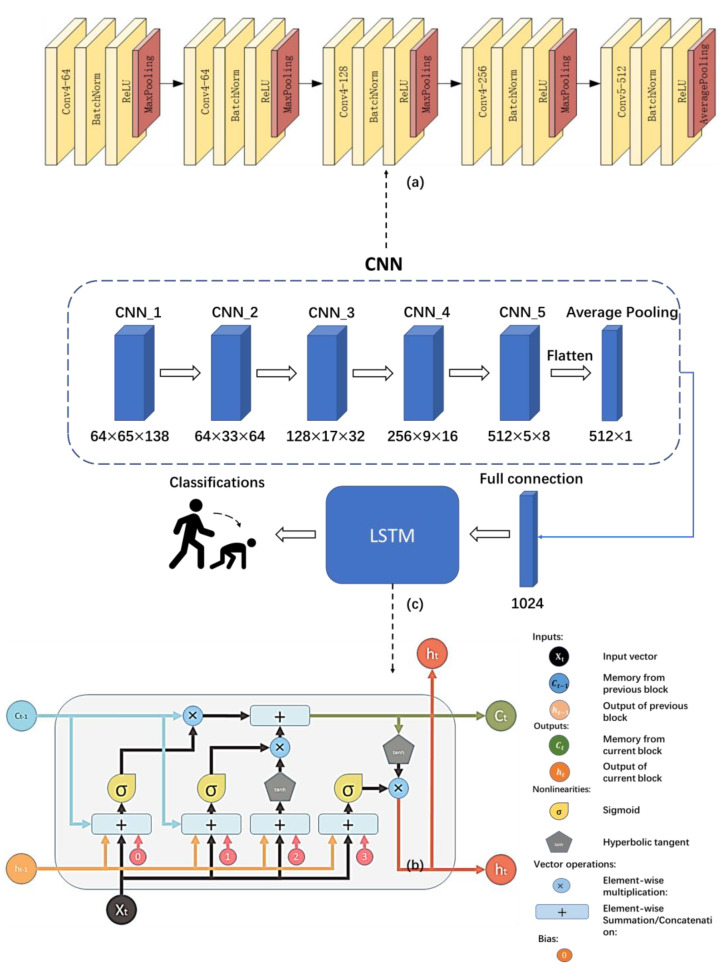
Structure of the model. Detailed structure of CNN layers in (**a**); The structure of LSTM in (**b**); The structure of the model combined by CNN and LSTM in (**c**).

As depicted in Figure 17c, the proposed model is based on a combination of CNN and LSTM. The structure of the CNN, shown in Figure 17a, was incorporated in the proposed model due to its ability to extract low-level spatial features from the data. This CNN comprises five layers, each of which includes a Convolutional layer, Batch Normalization (BN), a Rectified Linear Unit (ReLU), and a Pooling layer. The first four layers utilized Max pooling, while only the last layer employed Average Pooling. The utilization of Average Pooling in the last layer better retained the background features in the image and transmitted them into the LSTM as compared to Max pooling. We added BN and ReLU to the model to prevent overfitting, as these methods can normalize and nonlinearly map the data. The architecture of this CNN not only enhances the generalization and representation ability, but also accelerates the model’s convergence. We combined LSTM with CNN due to its remarkable capacity for extracting high-level temporal features from the data. Unlike RNN, which struggles to handle long-time image sequences, LSTM learns information features about the relationship between each image sequence through the forget gate, input gate, and output gate. In Figure 17b, the information on the cell state $C_{t-1}$ propagates across the main channel. The hidden state $h_{t}$ and input $X_{t}$ at state t modify $C_{t}$ as appropriate, after which it is passed to the next state. Finally, the information of the hidden state $h_{t-1}$ utilizes the structure of gates in LSTM to modify the cell state and calculate the output, thus solving the problem of the RNN gradient vanishing and exploding through three gates.

#### 3.4.2. Multi-Sensor Data Fusion Algorithms

Multi-Sensor data fusion refers to combining data from multiple sensors to enhance the accuracy, reliability and generalization of an HAR system. By doing so, the issues caused by single-sensor data, including environmental limitations, can be reduced. In our research, we explored three distinct data fusion algorithms: data level fusion, feature level fusion, and decision level fusion, and conducted experiments to compare their performance.

(1) Data level fusion

**Figure 18:**
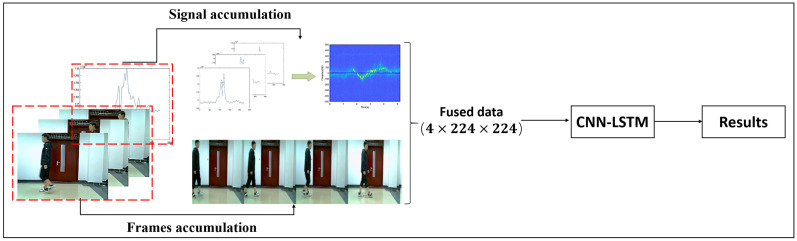
The block diagram of the data level fusion.

Data level fusion is a method that handles raw data at the bottom of the system with minimal data loss and maximum reliability. However, the performance of the data level fusion algorithm relies heavily on the type of sensor being used. If the sensors collect information that does not match, it becomes difficult to work with. Data level fusion combines different sensor data by using timestamp validation and channel stack. Once fused, the resulting data is input into the model for training and classification. Figure 18 shows the block diagram of the data level fusion process.

(2) Feature level fusion

Feature level fusion does not directly fuse the original data. Instead, this method extracts features from the data processed by each sensor and fuses the extracted features for recognition at the end of fusion. To accomplish this, we utilized two independent CNN networks: one CNN was used to extract radar spectrogram features, while the other CNN extracted image sequence features. The extracted feature maps were then combined by addition. After adjusting the size of these feature maps to ensure consistency, they were successfully fused in an LSTM network model.

The size of the fused feature map is represented by Equations (14) and (15):

(14) $$W^{\prime }=\frac{\left(W-F+2P\right)}{S}+1\;$$

(15) $$H^{\prime }=\frac{\left(H-F+2P\right)}{S}+1$$

in the equations presented, W and H represent the width and height of the feature map before convolution, while $W^{\prime }$ and $H^{\prime }$ are the width and height after convolution. The size of the convolution kernel is represented by F×F. P refers to padding and S represents stride.

(a) Feature addition

Before feature maps can be added to one another, they must first be converted to the same size and data type. Feature values are then added together one-by-one. Upon completion, the size and dimension of the feature map remain unchanged. If the extracted feature map *A* has a size of (W,H,D), then the size of feature map *B* must also be (W,H,D). The resulting fused feature map is represented by Equation (16).

(16) $$y_{i,j,d}^{Sum}=x_{i,j,d}^{A}+x_{i,j,d}^{B}\;$$

In Equation (16), where 1≤i≤W, 1≤j≤H and 1≤d≤D, $y^{Sum}$ represents the feature value in the fused feature map, while $x^{A}$ and $x^{B}$ represent the feature values of feature maps *A* and *B* at point (i,j,d), respectively.

(b) Feature concatenation

Feature concatenation does not require feature maps to have matching dimensions. This method concatenates feature maps along a specific data dimension, making it more suitable for fusing data from different modes or with different dimensions. Suppose the size of feature maps *A* and *B* are both (W,H,D), then the resulting fused feature map values are represented by Equation (17).

(17) $$y_{i,j,2d}^{concat}=x_{i,j,d}^{A}\;concat\;x_{i,j,d}^{B}\;$$

In Equation (17), where 1≤i≤W,1≤j≤H, and 1≤d≤D, $\;y^{concatenation}$ represents the feature value in the fused feature map. $x^{A}$ and $x^{B}$ represent the feature values of feature maps *A* and *B* at point (i,j,d), respectively. The new dimension after concatenation is represented by 2d.

**Figure 19:**
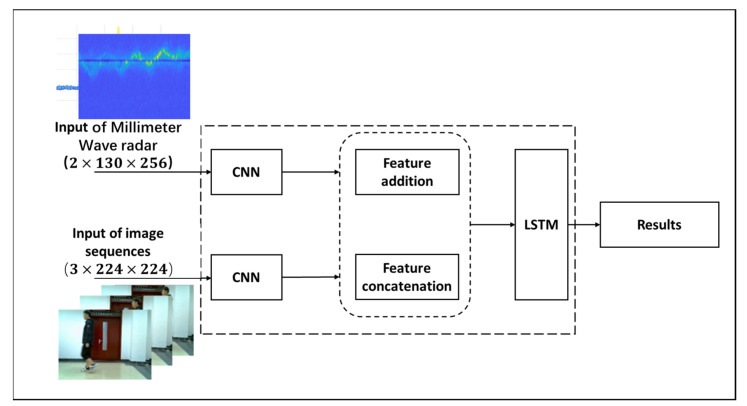
The block diagram of the feature level fusion.

As shown in Figure 19, the block diagram for feature level fusion is presented.

(3) Decision level Fusion algorithm

**Figure 20:**
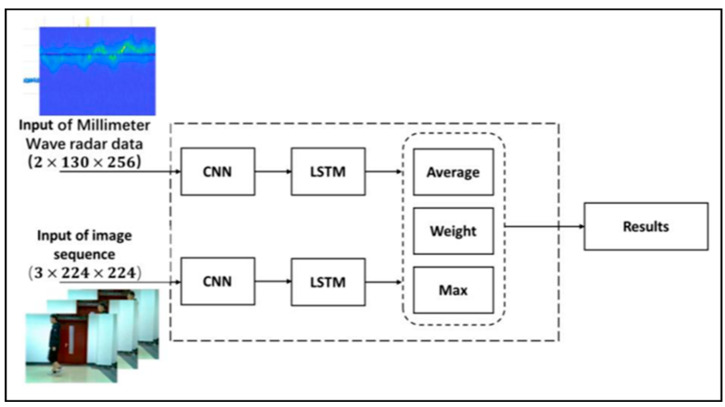
The block diagram of decision level fusion.

The decision level fusion algorithm differs from the other two algorithms in that it processes results output by each sensor at the end of the model. This allows for decision fusion to be more widely applicable to different types of sensors. After independently processing collected data, each sensor inputs its result into the decision module. The decision section then assesses these values by calculating the mean value, maximum value, distinguishing the contribution of each sensor through weighting, and other methods. The final output value is the result of classification. Figure 20 illustrates the block diagram for the decision level fusion process.

(a) Decision level average fusion (DLAF)

The average value provides an intuitive reflection of the comprehensive information contained within a set of data. While DLAF can take into account each sensor’s contribution to the output prediction results, any change to a single value will cause the average value to fluctuate. Additionally, the average value is more likely to be affected by extreme data groups that contain the maximum and minimum values. Equation (18) shows the output prediction results after applying DLAF.

(18) $$y\left(x\right)=\frac{1}{n}\sum_{i=1}^{n}g\left(x_{i}\right)$$

n represents the number of sensors, $g\left(x_{i}\right)$ represents the input value transmitted from each sensor to the average fusion module, and y(x) represents the final classification result.

(b) Decision-level weights fusion (DLWF)

It is well-known that the importance of each sensor may not be equal. In DLWF, different weights are assigned to the results of different sensor outputs in order to distinguish their individual contributions to the system. The formula for DLWF is shown in Equation (19).

(19) $$y\left(x\right)=\sum_{i=1}^{n}w_{i}g\left(x_{i}\right)$$

In Equation (19), $g\left(x_{i}\right)$ represents the input value of each sensor to the decision module, $w_{i}$ represents the weight assigned to the output of each sensor, and y(x) represents the final classification result. In addition, in order to ensure the validity of the weights, all sensors are considered as a whole. As such, the range of the weight assigned to each sensor is $0<w_{i}<1$ (with at least two sensors required for this method to work), and the sum of all weights must equal one ($\sum_{i=1}^{n}w_{i}=1$).

(c) Decision level maximum fusion (DLMF)

Each sensor’s output value already represents the classification result for its corresponding module. DLMF assigns a weight of 1 to the sensor with the highest predicted probability. For example, if Sensor A has an output prediction result of $\left\{p_{1},p_{2},p_{3},p_{4},\ldots ,p_{n-1},p_{n}\right\}$ and Sensor B has an output prediction result of $\left\{p_{1}^{\prime },p_{2}^{\prime },p_{3}^{\prime },p_{4}^{\prime },\ldots ,p_{n-1}^{\prime },p_{n}^{\prime }\right\}$, then the output of DLMF is shown in Equation (20).

(20) $$P\;=\max\left(\left\{p_{1},p_{2},p_{3},p_{4},\ldots ,p_{n-1},p_{n}\right\},\;\left\{p_{1}^{\prime },p_{2}^{\prime },p_{3}^{\prime },p_{4}^{\prime },\ldots ,p_{n-1}^{\prime },p_{n}^{\prime }\right\}\right),1<i\leq n$$

## 4. System Test

### 4.1. Experimental Data Collection

Experimental data was collected with permission from the volunteers. The group consisted of six males and four females, ranging in height from 159 cm to 189 cm, weights from 47 kg to 105 kg, and ages between 24 and 26. The volunteers performed five activities: sitting, squatting, walking, bending, and falling, with each repeated 30 times in normal and low-light environments. We ultimately collected a dataset comprised of 3000 combinations of radar and camera data sequences. This dataset was divided into a training set and a test set in an 8:2 ratio. More information about the dataset is shown in Table 5.

### 4.2. Evaluation Metrics

To assess the performance of our algorithm and network structure, we utilized several evaluation metrics, including accuracy and confusion matrices.

(1) Accuracy

(21) $$Accuracy=\frac{TP+TN}{TP+FN+FP+TN}$$

True positive (*TP*), true negative (*TN*), false negative (*FN*), and false positive (*FP*) are important metrics in evaluating the performance of a model. While *TP* represents the number of true positives, *TN* indicates the number of true negatives, *FN* is indicative of the number of false negatives, and *FP* represents the number of false positives. *Accuracy* is one of the key indicators used to evaluate the performance of a model, with a higher accuracy value indicating better performance.

(2) Confusion Matrix

The Confusion Matrix is often utilized to evaluate the quality of a classification model. The abscissa represents the true label, while the ordinate represents the predicted label. A greater number of predicted values distributed diagonally across the confusion matrix indicates better model performance.

### 4.3. Experimental Results

In this section, the proposed CNN-LSTM model was employed to compare its classification performance with traditional CNN and RNN models under different input data conditions. We also conducted an algorithm comparison experiment focused on the application of different fusion algorithms on the CNN-LSTM model. Different data fusion methods were evaluated using criteria such as accuracy, confusion matrix, and ROC curve. 

#### 4.3.1. Model Comparison

To evaluate the performance of the proposed CNN-LSTM model, we conducted a comparative analysis with traditional CNN and RNN models. Additionally, we compared the recognition performance of these models in HAR using different types of input data. Given that radar data is unaffected by lighting conditions, we exclusively used radar data collected in low-light environments for comparative experiments. Conversely, we compared camera data recorded in normal and low-light environments.

Table 6 shows that when millimeter-wave radar data is used as the model input, CNN, RNN, and CNN-LSTM models generally exhibited lower accuracy in HAR than camera data recorded under normal lighting conditions. However, in low-light environments, millimeter-wave data outperformed camera data, confirming that millimeter-wave radar is more appropriate for identifying human activities in such settings. The results of the radar data analysis indicated that the CNN-LSTM model performed best among the three models, with the RNN model exhibiting the lowest performance. This is mainly due to the suitability of the RNN model for extracting time series features from data, whereas it struggles to process spectrogram information. Furthermore, all three models demonstrated excellent HAR accuracy for camera data captured in normal lighting environments, with the recognition accuracy for all five activities exceeding 80%. Notably, the CNN-LSTM model exhibited the best performance, achieving recognition accuracy higher than 96% for all five activities, indicating the superior performance of this model for HAR.

To better demonstrate the classification effects of camera and millimeter-wave radar on HAR in low-light environments, we present the confusion matrix for camera and millimeter-wave radar data in Table 7 and Table 8, respectively. The values in the rows of the table represent predicted labels, while the vertical axis represents true labels. The main diagonal of the table represents accurately predicted values, and they are in bold and are highlighted with a gray background.

In order to demonstrate the misclassification of the combination of different low-light data with the CNN-LSTM model, Table 7 and Table 8 show the HAR results of low-light camera data and radar data, respectively.

Table 7 shows that the CNN-LSTM model struggles to accurately classify activities when using low-light camera data as the input. The classification accuracy for the five activities ranges from 49% to 63%. Sitting has the highest accuracy, because changes in body posture are most prominent compared to other activities. Additionally, sitting relies on external objects, such as the chair, which provides more features for recognition. Squatting has the lowest accuracy of HAR due to its similarity with sitting, which both involve a change in leg position. However, squatting lacks reference points that could provide additional features for HAR. Falling and bending are also commonly misclassified, as there is a significant amplitude change in the body’s trunk during falling, while bending exhibits a similar phenomenon, resulting in frequent misclassification between them. The result shows that the method based on low-light camera data combined with a model is difficult in HAR, and this method also leads to a large number of misclassifications between confused activities.

Radar signals are not affected by lighting conditions. Table 8 shows that combining radar data with a model has significantly improved the overall accuracy of HAR for five activities compared to using low-light camera data. The method achieved a recognition accuracy of 100% for walking, which is the highest among all activities. This is because the distinct signal features of limb swinging during walking can be accurately classified, distinguishing it from other activities. The result shows that the squatting activity had the lowest accuracy of 78%, with a misclassification rate of 16% which was interpreted as sitting due to similar signal features in leg and arm movement. The HAR accuracy improved when millimeter-wave radar data was used instead of camera data in low-light environments, resulting in lower overall activity misclassification rates. In Table 8, the highest misclassification rate is 16% for misclassifying squatting as sitting, followed by 8% for misclassifying falling as squatting or walking. Given the stable performance of millimeter-wave radar in low-light environments, we aim to improve HAR accuracy and reduce activity misclassification rates by combining fusion algorithms with fusion data.

#### 4.3.2. Algorithm Comparison

Our study aimed to enhance the performance of AI models in HAR under low-light environments, and we therefore utilized a data set recorded in such conditions for our experiments. Given that the proposed CNN-LSTM model outperformed both CNN and RNN models in HAR, we solely conducted comparative experiments on data fusion algorithms with the CNN-LSTM model, as shown in Table 9.

Table 9 demonstrates that the performance of the CNN-LSTM model significantly improves when fusion data is utilized as the model input in comparison to using camera or millimeter-wave radar data alone. In comparison to the HAR accuracy achieved by the CNN-LSTM model with camera data in low-light environments, the data level fusion algorithm, feature level fusion algorithm, and decision level fusion algorithm improve the accuracy by at least 26.68%, 19.87%, and 21.92%, respectively. Similarly, when compared to the CNN-LSTM model with millimeter-wave radar data in low-light environments, the performance of the CNN-LSTM model combined with fused data also shows great improvement. For instance, the accuracy of squatting and falling improves by 6.64% and 10.27%, respectively. Among the fusion algorithms, data level fusion achieves balanced accuracy for each activity, with an average recognition accuracy of approximately 95%, except for sitting, with a recognition accuracy of 94.55%. Although the recognition accuracy of feature addition and feature concatenation is higher than most single-input data in low-light environments, their overall recognition accuracy falls short of other fusion algorithms. The performance of all three decision level fusion algorithms surpasses that of the feature level fusion algorithms. Each decision level algorithm excels at recognizing specific activities; for example, DLAF has a bending accuracy of 99.12%, while DLMF achieves a falling accuracy of 98.98%. Overall, the data level fusion algorithm exhibits the best balanced performance, with an average recognition accuracy of each activity reaching approximately 95%. The experimental results demonstrate that the fusion algorithms combined with the CNN-LSTM model are effective in mitigating the problem of low HAR accuracy observed with single data inputs under low-light environments.

In order to demonstrate the misclassification of the combination of different low-light data with the CNN-LSTM model and different fusion algorithms, Table 10, Table 11 and Table 12 show the HAR results of data level fusion, feature level fusion, and decision level fusion, respectively. The values in the rows of the table represent predicted labels, while the vertical axis represents true labels. The main diagonal of the table represents accurately predicted values, and they are in bold and highlighted with a gray background.

According to Table 10, it can be seen that the HAR accuracy of the method based on the data-level fusion algorithm combined with fusion data is above 90%, with a maximum of 98% for walking and a minimum of 94% for bending. At the same time, the maximum misclassification rate is only 4%. From the results, it can be concluded that compared with the single low-light camera data and the single millimeter-wave radar data, this method has greatly improved both the HAR accuracy and the misclassification rate. The data-level fusion algorithm processes and fuses data collected by different types of sensors before the model training, which can better extract data features and improve HAR accuracy. Additionally, it is worth mentioning that this approach does not require any modifications of data features in the model and doesn’t involve any post-processing of output results. As a result, it is a method that can be easily implemented without requiring extensive work. 

The values in the rows of the table represent predicted labels, while the vertical axis represents true labels. The main diagonal of the table represents accurately predicted values, and they are in bold and highlighted with a gray background. Table 10 contains two confusion matrices, where the value on the left side of each cell represents feature addition and the value on the right side represents feature concatenation. The values with the high in bold represents better HAR accuracy, and the values with the low in bold represents a better misclassification rate.

The results presented in Table 11 indicate that the HAR accuracy of falling, sitting, and walking is higher, but the accuracy of bending is lower when feature addition is used as opposed to feature concatenation. From the misclassification, we can see that the feature addition method performs worse than the feature concatenation method. While concatenating features can increase dimensionality and improve training, it also amplifies the impact of noise in low-light camera data. On the other hand, feature addition maintains dimensionality but enhances motion features by adding feature values from different types of data. The findings suggest that both feature level fusion algorithms are superior to the single low-light camera data, and both perform slightly better than the single millimeter wave radar data. However, these methods fall short of the performance achieved by data level fusion.

The values in the rows of the table represent predicted labels, while the vertical axis represents true labels. The main diagonal of the table represents accurately predicted values, and they are in bold and highlighted with a gray background. Table 12 contains three confusion matrices, and from they are DALF, DLWF and DLMF from left to right. The values with the high in bold represents better HAR accuracy, and the values with the low in bold represents better misclassification rate.

In terms of HAR accuracy, DLWF has the three highest accuracies of 99%, 93% and 94% for falling, sitting and squatting, respectively, while DALF has the two highest accuracies of 99% for bending and walking. Referring to the misclassification rates, it appears that both DLWF and DALF have lower rates than DLMF, with both rates being below 10%. The highest misclassification rate of 11% is attributed to DLMF misclassifying squatting as sitting. DLWF intervenes in the importance of different types of sensors in the system by weighting final output of the classification values. The DALF averages the classification values obtained from different types of sensor data to achieve more balanced results and improves HAR accuracy. DLMF performs poorly among the three decision level algorithms. It chooses the classification value with the largest output, which may select the classification that best represents the activity, but there is also a high risk of selecting a classification with interference, resulting in low and unstable accuracy. This is why the misclassification rate of this method is the highest among the three algorithms. Decision-level fusion algorithms outperform single low-light camera data, single millimeter-wave radar data, and feature level fusion algorithms. The combination of three decision level algorithms with fusion data improves recognition accuracy and reduces the misclassification rates of some activities, particularly the DLWF and DALF. It is difficult to extract features from both low-light camera data and millimeter-wave radar data, making it challenging to effectively train the model. However, decision level fusion algorithms operate on classification values at the model end instead of the feature extraction module, making it easier to improve performance. However, the performance of decision level fusion algorithms is worse than that of data level fusion algorithms. Data level fusion algorithms process and fuse data at the top of the model, which better meets the input and feature extraction requirements of the model, and has lower complexity than decision level fusion algorithms.

## 5. Summary

We proposed an HAR system based on multisensor data fusion and the CNN-LSTM model to improve the accuracy of human activity recognition in low-light environments. Our multi-sensor acquisition system, consisting of a Logi C270 USB camera and TI 60GHZ IWR6843 millimeter wave radar, was calibrated from both spatial and temporal aspects to ensure effective data fusion. We preprocessed radar data to a time-frequency spectrogram and a range spectrogram, and camera data to image sequence. We trained and tested our proposed CNN-LSTM model on a dataset of 3000 samples collected from our multi-sensor acquisition system. The experimental results show that the CNN-LSTM model performs significantly better than traditional CNN and RNN models when using camera data under normal light and millimeter-wave radar as the model input. We compared three fusion algorithms and verified the effect on the CNN-LSTM model. The comparative experimental results show that the data level fusion algorithm is the most balanced and suitable for the system. Compared with camera data in low-light environments, the proposed system improves the HAR accuracy by at least 26.68%, and reduces the misclassification rate to 2~6%. However, there are still certain limitations in our work. Although the system is a multisensor one, and we can use millimeter-wave radar in HAR, thus avoiding the use of cameras in places with high privacy, the presence of cameras still reduces people’s comfort levels. Our model is trained with normal light camera data combined with radar data, but was tested with low-light data. We tried to train the model by using low-light data, but the results were not ideal. In the next work, we will assess the model’s ability to generalize and adapt to different lighting conditions, which is crucial for the practical applications of HAR. Furthermore, it is important to note that the volunteers participating in data collection were all young people, whose activities may not be entirely representative of those of the elderly. Therefore, collecting and analyzing data on the actual activities of the elderly in real-life settings is a challenging and worthwhile objective for future research. In future work, we will plan to further improve the accuracy of the model through feature extraction, data processing, and fusion algorithms, and to collect more human activity data to build larger datasets and improve the robustness of the model.

## Figures and Tables

**Table 1:** Parameters of Logi C270.

Number	Name	Parameter
1	Sensor	CMOS
2	Pixel	3 million
3	Capture Size	1280 × 720
4	Resolution	1280 × 720
5	Max FPS	30 frames/s
6	Interface	USB 2.0

**Table 2:** Components of the IWR6843ISK.

Name	Introduction
Antenna	AN7105 dual-polarized patch antenna
RF front-end chip	AWR1843, supports frequency ranges from 60 GHz to 64 GHz
Digital signal processor chip	Uses high-performance floating-point DSP architecture
Microcontroller	RM57L843, uses ARM Cortex-R5F architecture

**Table 3:** Parameters of the IWR6843ISK.

Number	Name	Parameter
1	Types	FMCW
2	Tuning Frequency	60–64 GHz
3	Number of Receivers	4
4	Number of Transmitter	3
5	Azimuth FOV (deg)	±60
6	Azimuth Angular Resolution (deg)	15
7	Elevation FOV (deg)	±15
8	Elevation Angular Resolution (deg)	58
9	Arm CPU	ARM R4F @ 200 MHz
10	Memory (kb)	1792

**Table 4:** Specification and Performance Parameters of the MMWAVEICBOOST.

Specification	Performance Parameters
Operating frequency	76 GHz to 81 GHz
Receiver sensitivity	−80 dBm
Ranging range	maximum 8 m
Field of view (FOV)	60 degrees (horizontal) × 20 degrees (vertical)
Data output	Distance, speed, angle, target information, etc.

**Table 5:** Division of datasets.

Environment	Proportion	Activity	(Quantity, Proportion)
Normal light	Train set (1200, 40%) Test set (300, 10%)	Sitting	(300, 10%)
		Squatting	(300, 10%)
		Walking	(300, 10%)
		Bending	(300, 10%)
		Falling	(300, 10%)
Low-light	Train set (1200, 40%) Test set (300, 10%)	Sitting	(300, 10%)
		Squatting	(300, 10%)
		Walking	(300, 10%)
		Bending	(300, 10%)
		Falling	(300, 10%)

**Table 6:** Accuracy of different models on human activity recognition.

Sensor	Input Data	Environment	Model	Activities				
				Sitting	Bending	Walking	Squatting	Falling
Radar	Spectrogram	Low-light	CNN	78.94%	89.74%	97.37%	77.50%	78.94%
			RNN	33.92%	73.62%	17.21%	12.58%	31.56%
			CNN-LSTM	95.83%	89.29%	100.00%	78.12%	80.00%
Camera	Image sequence	Normal light	CNN	94.48%	91.36%	98.87%	85.62%	98.23%
			RNN	92.58%	94.27%	98.91%	80.23%	93.41%
			CNN-LSTM	98.26%	97.87%	100.00%	96.12%	98.24%
	Image sequence	Low-light	CNN	58.64%	39.59%	58.62%	36.87%	40.12%
			RNN	70.53%	43.51%	52.09%	77.50%	62.91%
			CNN-LSTM	63.17%	57.36%	53.37%	12.58%	69.23%

**Table 7:** The confusion matrix of the CNN-LSTM model with camera data in low-light (unit: %).

	Predict	Bending	Falling	Sitting	Squatting	Walking
True						
Bending		57	21	11	4	6
Falling		10	57	12	6	2
Sitting		9	13	63	2	4
Squatting		9	13	21	49	5
Walking		11	9	14	13	54

**Table 8:** The confusion matrix of the CNN-LSTM model with radar data in low-light (unit: %).

	Predict	Bending	Falling	Sitting	Squatting	Walking
True						
Bending		89	0	4	7	0
Falling		0	80	4	8	8
Sitting		0	0	96	4	0
Squatting		3	3	16	78	0
Walking		0	0	0	0	100

**Table 9:** Accuracy of CNN-LSTM models under different fusion algorithms (low-light environments).

Algorithm		Activities				
		Sitting	Bending	Walking	Squatting	Falling
Data level fusion		94.55%	94.12%	98.04%	95.92%	95.91%
Feature level fusion	Addition	86.96%	89.11%	90.53%	84.76%	93.52%
	Concatenation	83.04%	92.38%	86.96%	85.29%	90.27%
Decision level fusion	DLAF	91.26%	99.12%	98.96%	92.11%	96.94%
	DLWF	89.29%	95.24%	91.30%	86.27%	91.15%
	DLMF	93.20%	97.35%	97.92%	93.86%	98.98%

**Table 10:** Confusion matrix of data level algorithm (unit: %).

	Predict	Bending	Falling	Sitting	Squatting	Walking
True						
Bending		94	2	4	0	0
Falling		0	96	4	0	0
Sitting		0	4	95	2	0
Squatting		0	2	2	96	0
Walking		0	0	0	2	98

**Table 11:** Confusion matrix of feature addition and the feature concatenation algorithm (unit:%).

	Predict	Bending	Falling	Sitting	Squatting	Walking
True						
Bending		89/ **92**	5/**4**	4/4	4/**0**	0/**0**
Falling		**1**/5	**94** /90	**1**/2	5/**2**	**0**/1
Sitting		**1**/2	2/**1**	**87** /83	**10**/12	**0**/13
Squatting		0/0	**1**/2	**1**/12	85/85	3/**1**
Walking		**1**/2	2/2	**3**/5	3/3	**91**/87

**Table 12:** Confusion matrix of the DALF, DLWF and DLMF algorithms (unit:%).

	Predict	Bending	Falling	Sitting	Squatting	Walking
True						
Bending		**99** /97/95	**0**/**0**/3	**1**/2/**1**	**0**/1/1	0/0/0
Falling		2/**0**/5	97/ **99** /91	**0**/**0**/3	1/**0**/**0**	**0**/1/1
Sitting		1/1/1	0/0/0	91/ **93** /89	6/**5**/7	2/**1**/3
Squatting		0/0/0	1/**0**/**0**	**1**/6/11	92/ **94** /86	**0**/**0**/3
Walking		**0**/**0**/2	**0**/1/1	**0**/1/5	1/**0**/**0**	**99** /98/86

## Data Availability

The data is not publicly available in order to maintain the privacy of the volunteers.
